# New technique for direct targeting of the ventral intermediate nucleus using magnetic resonance-guided focused ultrasound

**DOI:** 10.3389/fradi.2025.1588379

**Published:** 2025-06-11

**Authors:** Juan Ángel Aibar-Duran, Rodrigo Akira Watanabe, Nathan J. McDannold, G. Rees Cosgrove

**Affiliations:** ^1^ Department of Neurosurgery, Hospital de la Santa Creu i Sant Pau, Barcelona, Spain; ^2^ Department of Neurosurgery, Federal University of São Paulo, São Paulo, Brazil; ^3^ Department of Radiology, Mass General Brigham, Boston, MA, United States; ^4^ Department of Neurosurgery, Mass General Brigham, Boston, MA, United States

**Keywords:** magnetic resonance guided focus-ultrasound, ventral intermediate nucleus, direct targeting, white matter null, thalamotomy, essential tremor

## Abstract

**Background:**

Accurate targeting and lesion placement are critical in treating movement disorders with magnetic resonance-guided focused ultrasound (MRgFUS). Indirect atlas-based targeting often lacks precision. Direct anatomical targeting with 3T MRI offers a promising alternative. This report aims to refine MRgFUS thalamotomy by integrating advanced imaging and lesion conformality strategies.

**Material and methods:**

Preoperative and postoperative white matter null (WMn) MR-imaging was employed for direct Vim detection. Essential anatomical landmarks are identified. Dual-lesion conformality was used to adapt to the spatial distribution of the Vim.

**Results:**

Lesions of the Vim were identifiable using the postoperative WMn MRI sequence. The direct visualization of the Vim usually avoids extension into the internal capsule and the sensory thalamic nucleus. Sagittal imaging confirmed the dual-lesion conformational strategy which conforms to the antero-superior orientation of the Vim.

**Conclusions:**

Direct Vim targeting for MRgFUS is feasible for individual cases with the use of WMnMPRAGE MRI sequences. The use of lesion conformality adapts well to the anatomical and spatial distribution of Vim. Further studies will be needed to confirm the safety profile of this approach and correlate with clinical outcomes.

## Introduction

Successful magnetic resonance-guided focused ultrasound (MRgFUS) thalamotomy requires accurate targeting and conformal thermal lesioning of the target volume to provide enduring tremor control and minimize side effects. While protocols may vary (1), there is a general consensus that MRgFUS targeting the ventral intermediate nucleus (Vim) is both effective and safe for treating patients with essential tremor and tremor-predominant Parkinson's disease (2–4). Precise targeting and lesion conformality—including accurate location, size, shape and extent—are critical for optimizing clinical outcomes and reducing the risk of complications (5–7).

**Figure 1:**
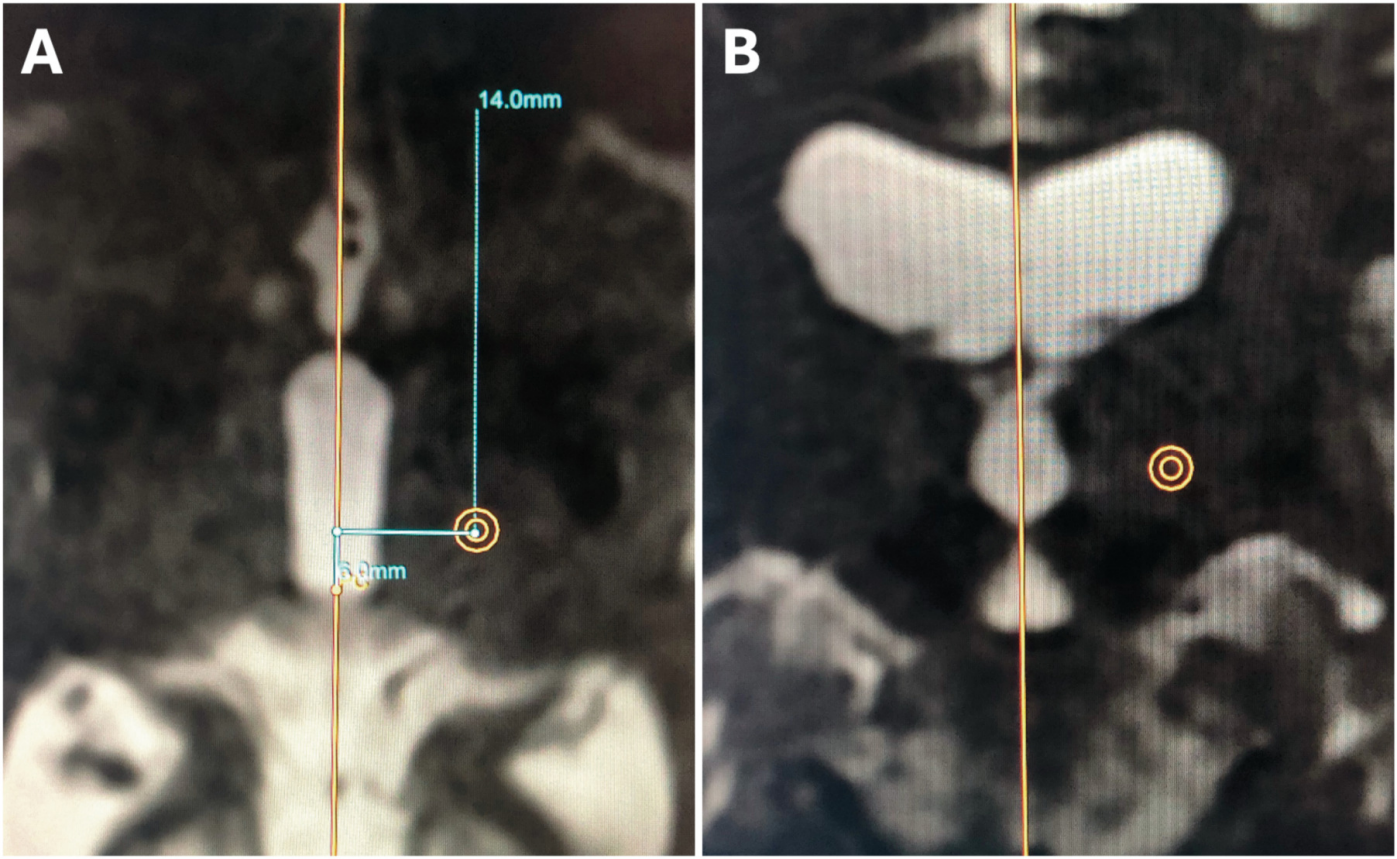
Atlas-based targeting using standard coordinates on a T2w MRI sequence **(A**: axial viw; **B**: coronal view). Note the complete lack of anatomical landmarks of any intra-thalamic or extra-thalamic structures.

MRgFUS is typically performed using indirect coordinates to localize the Vim. The intra-operative MRI obtained with the patient in the Insightec FUS treatment helmet is used to identify the anterior commissure (AC) and posterior commissure (PC) and then by selecting a target ¼ distance of the AC/PC length in front of PC and 14 mms from the midline. These images provide very limited visualization of the thalamus or its surrounding structures (Figure 1).

Although direct visualization of the Vim and other thalamic nuclei has been achieved using 7T MRI, this technology is not yet widely available in clinical practice (5, 8). Thalamic atlas models developed in Montreal Neurological Institute (MNI) space, are valuable for group-level analysis but less applicable to individual cases due to differences in brain morphology (5, 8). To ensure the reproducible application of MRgFUS, a straightforward, individualized, and direct anatomical targeting approach using standard 3T MRI is required.

In this report, we detail the key 3T MRI sequences for direct Vim targeting, highlighting its essential anatomical landmarks. Additionally, we discuss the technical nuances of our conformal lesioning strategy, optimized to cover the Vim in its supero-anterior orientation within the thalamus.

## Materials and methods

Since the launch of our MRIgFUS program for movement disorders, the targeting strategy at our center has evolved significantly. Initially, we relied on indirect coordinate-based targeting of the Vim, a classical approach applied in approximately 400 cases. More recently, in the latest 200 cases, we have shifted to a refined strategy that involves visual localization of the Vim—either directly or indirectly through surrounding anatomical landmarks.

To improve the visualization of Vim, we obtain a pre-operative MRI which includes standard anatomical and DTI sequences (32 directions, b = 1000s/mm^2^), as well as a white matter null magnetization prepared rapid gradient echo (WMnMPRAGE) sequence used for targeting. This sequence uses the following scan parameters: excitation repetition time (TR): 4000.0 ms, inversion time (TI): 500 ms, echo time (TE): 3.82 ms, bandwidth (BW): ±31.25 kHz, flip angle (FA): 7, field-of-view (FOV): 224 mm, slice thickness: 1.0 mm, slice number: 160, scan time: 7:00 min (9). These scans are co-registered to the patients pre-operative CT to allow for intra-operative target selection. All imaging is performed on a Siemens Magnetom 3.0T XR Numaris MRI scanner.

Pre-op CT were done 3–6 months in advance; pre-op MRI was performed 24 h before the procedure. CT and MRI were co-registered and loaded into the treatment console. During the procedure, a standard FIESTA MRI series was acquired and aligned with the pre-op images for AC-PC targeting. Target coordinates are first selected using standard atlas-based coordinates and adjusted using WMnMPRAGE images that offer direct visualization of the Vim.

### Imaging data and anatomical landmarks

**Figure 2:**
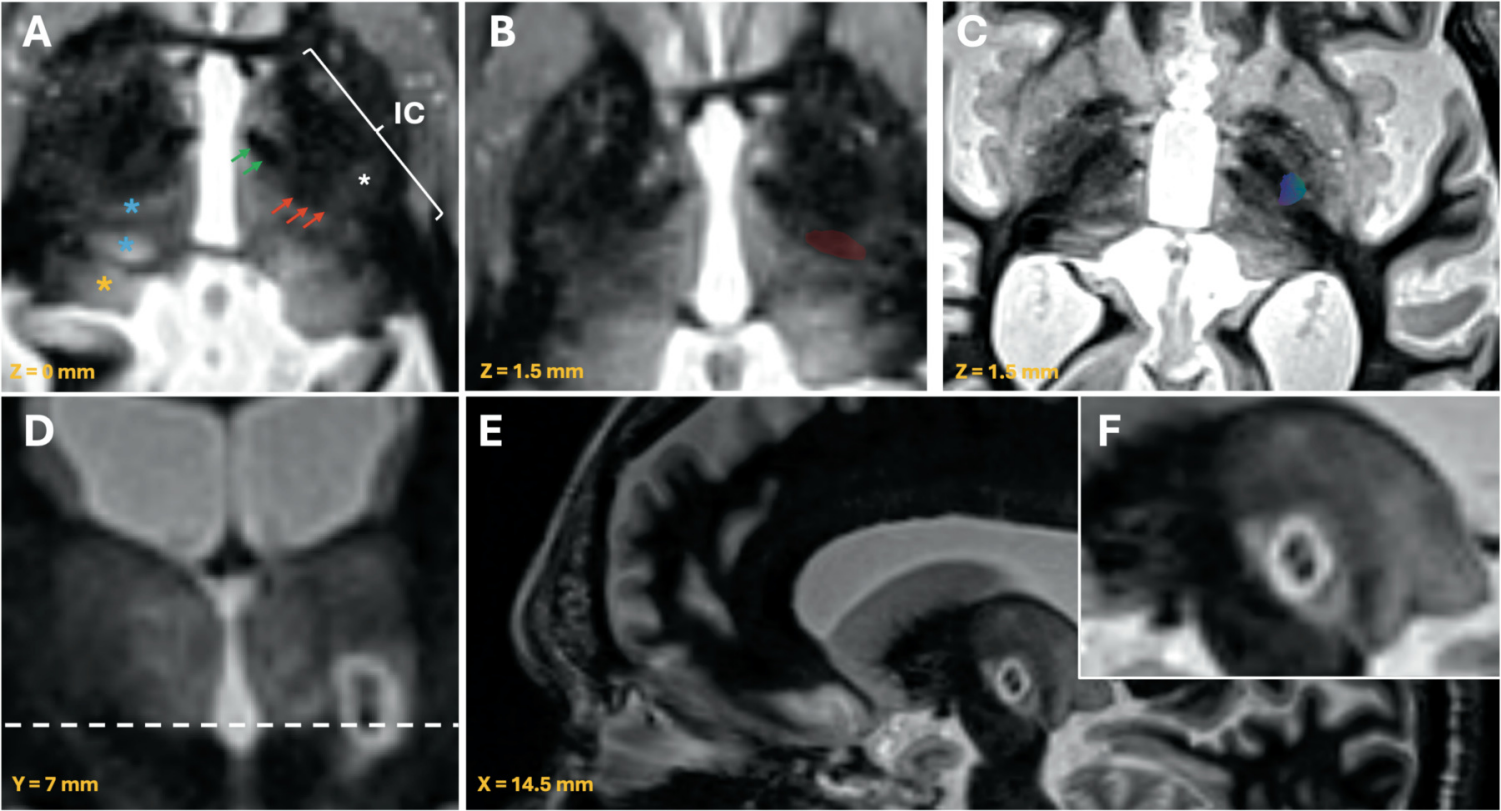
Multi-panel figure illustrating key anatomical landmarks (**A–C)** and significant postoperative outcomes **(D–F)** on WMnMPRAGE sequence. **(A)** Axial WMnMPRAGE sequence showing the dentatorubrothalamic tract (DRTT) (red arrows) located slightly ventral to its entry into the ventral intermediate nucleus (VIM). Since direct visualization is being proposed, it is likely that this corresponds to the larger portion of the DRTT—namely, the decussating (indirect) bundle. The mammillothalamic fascicle (MTF) (green arrows) is visible anterior and medial to the DRTT. The internal capsule (IC) is depicted, and within it, the projections fibers from the corticospinal tract (white asterisk). On the lest thalamus the sensory nuclei (blue asterisks) and the pulvinar (orange asterisk) are visible. **(B)** Delineation of the Vim, always sitting above the DRTT and slightly medial to the internal capsule hyperintensity. **(C)** Tractography computation of the corticospinal tract, corresponding to the hyperintense structure within the posterior limb of the IC. **(D)** Coronal view highlighting the superior-inferior extension of the acute lesion (24 h postoperative MRI). Notably, the lesion (zone I/II) extends to the intercommissural plane (white discontinuous line). **(E)** Sagittal image showing the anterior-superior tilted orientation of the lesioning strategy, mimicking the spatial organization of VIM. **(F)** Detail of the dual-lesion strategy within the inferior and medium part of the VIM. Tract reconstruction was performed using a deterministic approach in Brainlab Elements® with an angular threshold of 45° and fractional anisotropy of 0.2 cutoff values.

Visual analysis in the axial plane of the WMnMPRAGE sequence (Figure 2A) reveals several intra-thalamic anatomical landmarks useful for localizing the Vim. Among the most important is the entering dentatorubrothalamic tract (DRTT), appearing as a distinct ovoid hypo-intensity in the posterior third of the thalamic mass. Another useful anatomical reference is the mammillothalamic tract (MTT), located anterior and medial to the DRTT. Generally, the division between the anterolateral nuclei (VA, Voa, Vop, Vim, VPL, VPM) and the medial nuclei (Pf-MC complex, MD) are evident, as is the anterior margin of the pulvinar (Figures 2A,B).

Finally, the extra-thalamic landmark that can assist in Vim localization is the pyramidal tract (PT), which appears as a hyperintense band compared to the rest of the internal capsule fibers (asterisk on Figure 2A). The DRTT and the Vim are always located 2–3 mm medial to the medial border of this hyperintensity. Confirmation that the hyperintense band seen on the WMnMPRAGE sequence within the posterior limb of the internal capsule correlates with the cortico-spinal tract is shown using tractography computation (Figure 2C).

### Lesion conformality

Having identified the DRTT, Vim and their surrounding structures accurately, a lesioning strategy can be confidently adapted to provide a more 3D conformational thalamotomy. Once the DRTT is located at the intercommissural plane (z = 0 mm in Figure 2A), advancing 1.5–2.0 mm superiorly leads directly to the ventral portion of the Vim. Typical native coordinates for the first therapeutic sonication are as follows: x = 11 mm lateral to the wall of the third ventricle, y = 6.5–7.0 mm anterior to the posterior commissure, and z = 1.5–2.0 mm superior to the intercommissural plane. These coordinates are adjusted according to the anatomical and imaging considerations described above. Lesioning parameters are chosen with a high energy and a relatively short sonication duration to create a lesion @ 3–4 mm in diameter so that the lesion extends down to the most ventral aspect of Vim and just slightly into the zona incerta. Following an on-table examination to evaluate tremor reduction and potential side effects, a second therapeutic sonication is typically performed in a slightly more anterior and superior location (y = 7.5–8.0 mm anterior to the posterior commissure, and z = 2.5–3.0 mm superior to the inter-commissural plane), using higher energies and longer duration to create a lesion 4–5 mms in diameter. This lesioning strategy generally creates a final lesion that better conforms to the anatomic size and orientation of the Vim.

## Results

After applying this direct targeting strategy in 200 hundred cases, in approximately 70% of cases, the first lesion is placed 6.5–7 mm anterior to PC, 11 mm lateral to the wall of the lateral ventricle and at the level of the inter-commissural plane. In the remaining 30% of cases, these coordinates may vary by 2–5 mms. Regarding sonication parameters, energy levels varied between 14,000 and 25,000 Jules in most cases, lasting between 18 and 15 s. Generally three therapeutic sonications were performed (range of 2–6). Lesion sizes varied considerably depending on the number of therapeutic sonications. In most cases, termal lesions on the table measured approximately 4–6 mm along the coronal axis and 3–5 mm along the axial and sagittal axes.

Postoperative 24 h WMnMPRAGE MRI acquisition typically confirms lesion placement within Vim, along with the corresponding predicted clinical effects. Although clinical outcomes for this technique must be confirmed, the initial results suggest long term tremor improvement and an improved adverse effects profile with a consistent reduction in limb and labial paresthesia. The inferior margin of the lesion (zones I and II) generally extends slightly below the inter-commissural plane (Figure 2D). The shape and size of the lesion is better aligned with the three-dimensional layout of the Vim (Figures 2F,E), following its supero-anterior configuration. The size of these lesions is usually 6 × 8 × 5 mm^3^ (axial × coronal × sagittal), with some degree of overlap. After applying this methodology in >200 cases, the team has gained confidence in both the targeting and lesioning strategy.

## Discussion

The present report demonstrates the feasibility of utilizing a direct Vim targeting strategy in a clinical MRgFUS setting. This approach improves targeting accuracy by relying on identifiable anatomical landmarks within and around the thalamus. In addition, the lesioning strategy adapts better to the spatial conformation of the Vim.

The Vim's location within the thalamus is particularly sensitive, as it is adjacent to critical structures such as the pyramidal tract laterally, and the medial lemniscus and sensory thalamic nucleus posteriorly (10). Clinically, this proximity has been reflected in early MRgFUS studies, which reported a high incidence of paresthesiae and imbalance, most of which were temporary (2, 11).

Inter-institutional variability in indirect targeting strategies remains a significant challenge in MRgFUS thalamotomy. Differences in coordinate systems, imaging protocols, and anatomical reference points can lead to inconsistent Vim localization across centers. While some institutions rely on fixed stereotactic coordinates based on the anterior and posterior commissures (e.g., 25% of the AC–PC distance anterior to the PC), others incorporate patient-specific modifications or additional reference landmarks (12, 13). These variations are further compounded by differences in image resolution, segmentation software, and operator-dependent decisions. Moreover, the accuracy of indirect targeting may deteriorate in patients with anatomical distortions, such as ventricular enlargement or cerebral atrophy, where standard atlas-based coordinates may no longer correspond to the true location of the Vim. This lack of standardization underscores the importance of moving toward more individualized and anatomically grounded targeting approaches.

Many efforts have been made to accurately localize the Vim and clearly define its boundaries. The use of fiber-filtering techniques, such as dentatorubrothalamic tract (DRTT) tractography, has gained popularity as a means to optimize Vim targeting strategies (6). Theoretically, the best possible outcome—maximizing benefit while minimizing morbidity—would be achieved by identifying not only the DRTT, but also the pyramidal and somatosensory tracts (14). However, the methodologies for these computational techniques are not yet standardized, and their results may vary between centers or even depend on the operator's experience (e.g., key ROI selection, fractional anisotropy values, angle thresholds) (6, 14, 15). As presented in this work, direct targeting of the Vim—either through direct visualization or by identifying nearby anatomical landmarks, as described here—may offer significant advantages in individual cases. Integrating tractographic methods into this protocol could further enhance the precision of the targeging strategy. Nevertheless, direct anatomical targeting should be considered a complement—not a replacement—for intraoperative clinical exploration. Given the Vim's small size and inter-individual variability, real-time patient response during procedures like MRgFUS remains essential to refine target positioning and confirm therapeutic efficacy.

Alternative targeting strategies include the use of 7T MRI or 3D-redering of thalamis nuclei based on computational modeling (5, 8, 9, 16). Nevertheless, these analyses are typically derived from the combination of images from multiple subjects, and applying them reliably to individual cases remains a significant challenge. Recent efforts have highlighted the need for standardized neuroimaging approaches to the human thalamus, emphasizing the importance of harmonized segmentation protocols, validated ground truths, and open-source datasets to improve consistency across clinical and research settings (17). Such initiatives support the broader goal of achieving accurate and reproducible thalamic targeting, which is critical in procedures like MRgFUS.

**Figure 3:**
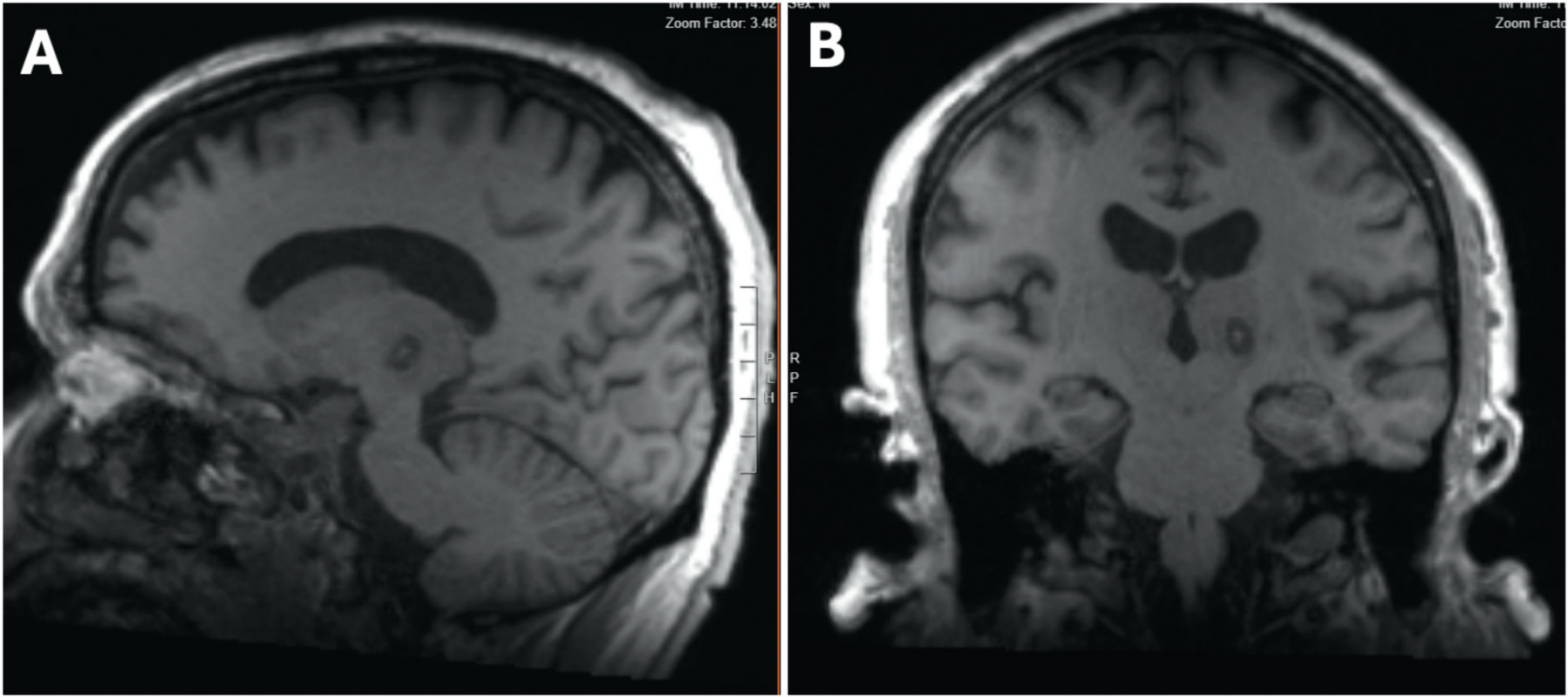
T1w postoperative MRI showing the conformational behavior of the standard lesioning strategy. Notably, on the sagittal view, the lesion is tilted in an oblique posterior angle **(A)** and in an oblique medial angle on the coronal view **(B)**, which do not follow the anatomical orientation of the Vim.

Because of phase shifting in ultrasound waves (18), the typical shape of the standard MRgFUS thermal lesion is an ovoid sphere with an orientation of its long axis from posterior to anterior and from medial to lateral. This geometry was in conflict with the natural orientation of the Vim and led to unwanted neurological side effects (Figure 3). Therefore, a lesioning strategy that adapted better to the Vim's anatomy was incorporated.

**Figure 4:**
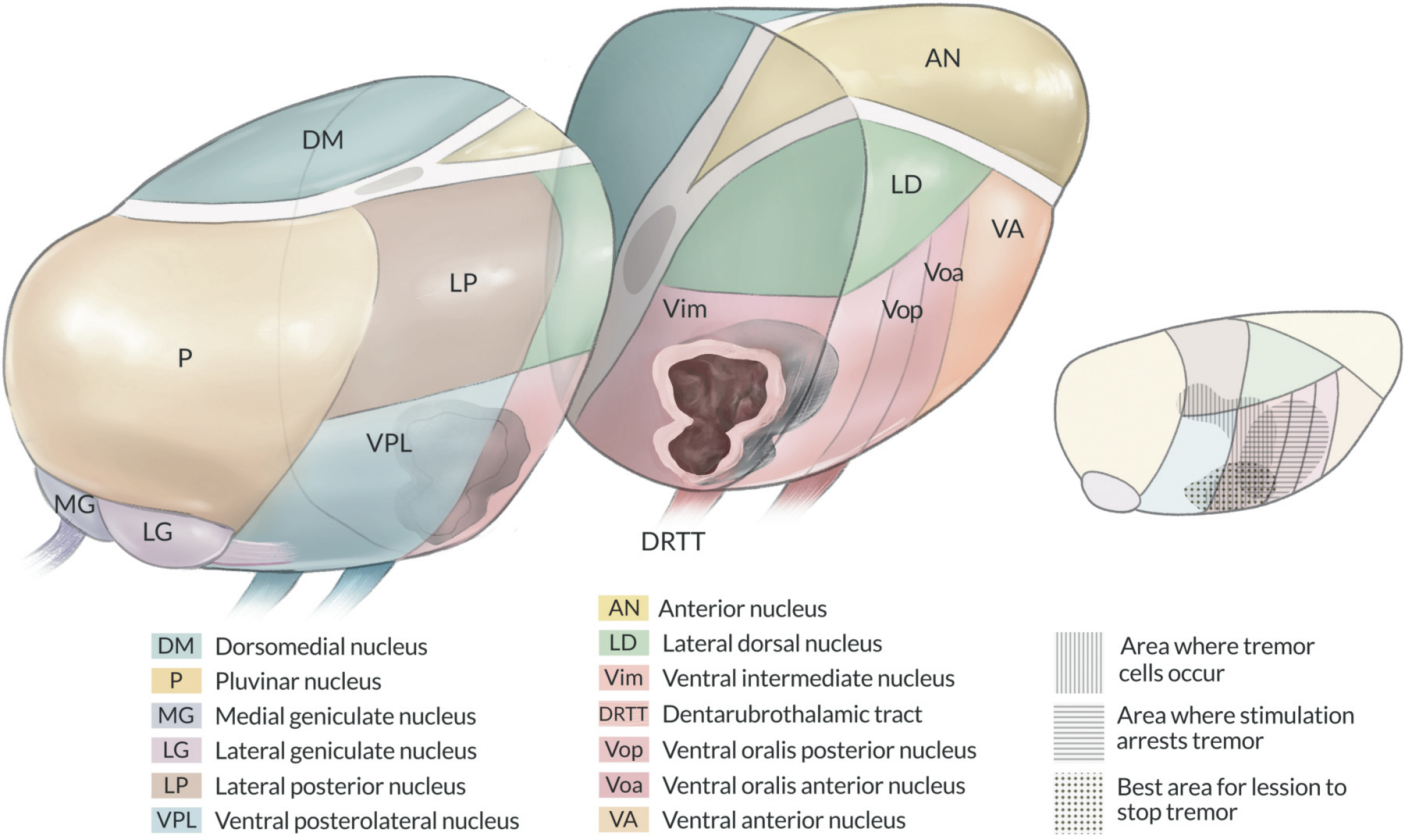
Original artwork illustrating the thalamic nucleus with detailed emphasis on the Vim. The Vim is shown running superiorly and anteriorly at an oblique angle toward the frontal lobe. The adaptation of the lesioning strategy to this anatomical configuration maximizes coverage of a critical Vim volume, which might minimize tremor recurrence.

Anatomically, the Vim tilts anteriorly and superiorly, following an oblique angle with the DRTT entering in its most ventral portion. Direct visualization of the Vim, combined with a lesion conformational strategy, enables precise targeting of the central mass of the nucleus. By maintaining the lesion within the boundaries of the VIM, neurological side effects could potentially be minimized. As recently reported, post-operative lesion size and extent into thalamic nuclei or surrounding regions (subthalamic area or internal capsule) might be key to predict adverse effects of the therapy (19). Figure 4 shows how the lesioning strategy adapts to the Vim's orientation, trying to catch a critical volume of the nucleus without affecting surrounding structures.

The placement of the first lesion slightly above the intercommissural plane has also been reported to produce a greater beneficial effect on tremor (7). While the clinical impact of the lesion conformality described here remains to be validated, the authors of this report believe that it could reduce unwanted side effects and contribute to reducing the rate of tremor recurrence by achieving a critical lesion volume within the Vim and its associated motor networks.

### Limitations

One limitation of this study lies in the potential imaging artifacts associated with the WMnMPRAGE sequence used for direct Vim targeting. Although the high isotropic resolution (1.0 × 1.0 × 1.0 mm^3^) and use of parallel imaging (GRAPPA, acceleration factor = 2) improve anatomical delineation and reduce acquisition time, minor blurring or partial volume effects may still occur, particularly at tissue interfaces. Signal uniformity can also be affected by B1 inhomogeneities; however, this was addressed using TrueForm B1 shimming and 2D distortion correction. Despite these mitigation strategies, subtle artifacts may influence boundary precision in some cases. Future refinements incorporating motion correction or complementary imaging contrasts may further enhance target visualization and segmentation accuracy.

## Conclusion

Direct Vim targeting for MRgFUS is feasible for individual cases with the use of WMnMPRAGE MRI sequences. The use of lesion conformality adapts well to the anatomical and spatial distribution of Vim. Further studies will be needed to confirm the safety profile of this approach and correlate with clinical outcomes.

## Data Availability

The original contributions presented in the study are included in the article/Supplementary Material, further inquiries can be directed to the corresponding author.
